# Heat Nests: The Impact of Climate Change on Loggerhead Turtle (*Caretta caretta*) Nesting Distribution in Sicily (Italy)

**DOI:** 10.1002/ece3.71177

**Published:** 2025-04-21

**Authors:** Chiara Siddiolo, Antonietta Rosso, Grazia Orecchio, Mario Lo Valvo

**Affiliations:** ^1^ University School for Advanced Studies IUSS Pavia Pavia Italy; ^2^ Department of Biological, Geological and Environmental Sciences University of Catania Catania Italy; ^3^ Dipartimento Scienze e Tecnologie Biologiche, Chimiche e Farmaceutiche University of Palermo Palermo Italy

**Keywords:** *Caretta caretta*, distribution, MaxEnt, Mediterranean, Seaturtle, Sicily

## Abstract

This study aims to update and establish a comprehensive list of nesting sites in Sicily and its minor islands, investigate the distribution and environmental suitability of the loggerhead sea turtles' nesting in Sicily using spatial distribution models (SDMs), and perform a gap analysis considering the protected area network in Sicily. Location: Sicily (Italy). Time period: 1979–2022. Data on Loggerhead seaturtle's nests were collected through several sources, including literature, monitoring records from WWF's Progetto Tartarughe, reports from the local fauna haunting, online articles, referrals on websites and social networks often related to monitoring activities. GIS was used to realize distribution maps. Bioclimatic indicators were downloaded through Copernicus Climate Change Service. Predictors were eventually projected on the WorldClim's dataset. Suitability distribution models (SDMs) were realized using the maximum entropy model (MaxEnt software). *Caretta caretta* 's nests distribution map and environmental suitability map were overlaid with the Natura 2000 sites map in Sicily. The results confirm that the main nesting areas are mostly concentrated along the southern and eastern coasts of Sicily, with increasing numbers observed over recent years. Additionally, global warming has made some beaches even more suitable along the north coast of the main island. The variable affecting this species the most is the Mean Temperature of the Coldest Quarter (Bio11). Overlaying nesting distribution and environmental suitability maps with Natura 2000 sites revealed significant portions of nests occurring outside protected areas, highlighting the need for expanded conservation efforts.The demographic increase of nesting events in Sicily is induced by a northward shift of the species distribution led by rising temperatures and probably due to climate change.

## Graphical abstract

To establish and update the list of nesting sites in Sicily and its minor islands. To investigate the Sicilian distribution and nesting suitability using spatial distribution models (SDMs). Performing a gap analysis in a GIS environment considering the protected area network in Sicily.

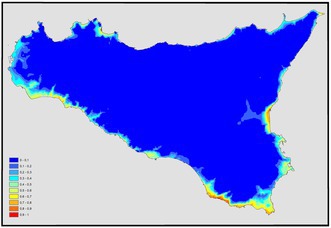

## 1 Introduction

The loggerhead sea turtle, *Caretta caretta* (Linnaeus, 1758), is the most common of the mediterranean sea turtles (Casale and Margaritoulis 2010), and it has a cosmopolitan distribution, nesting in the widest geographical range of any sea turtle. It inhabits the Atlantic, Indian, and Pacific Oceans and the Mediterranean Sea. In the Mediterranean Sea, this species has a wide nesting area along the coasts of Greece, Turkey, Cyprus, and Libya, with minor breeding sites across Egypt, Israel, Lebanon, Syria, and Tunisia, but also occasional nesting concerning Spain, France, Italy, and their surrounding islands (Casale et al. 2018).

The interest in loggerhead sea turtles in the Mediterranean, particularly in Italy, has constantly increased during the last four decades, and nesting sites were recorded on the coasts of Sicily, Sardinia, Apulia, and the Ionic coasts of the Basilicata and Calabria regions, where nesting is considered occasional, except for the Ionic areas of southern Calabria and the Pelagian Islands (Linosa and Lampedusa). As with every other sea turtle species, loggerheads usually nest in specific areas, and nest counts represent the most common index of the population's size (Casale and Tucker 2015; Mazaris et al. 2017).

There has been a significant increase in the number of recorded nests along the Italian shores over the years along with a growing number of deposition sites in Sicily. On the island, together with the increase of reported nests, the involvement of several coastal areas in addition to the Pelagian Islands, where nesting has always occurred, is increasing (www.legambiente.it). Among the living tetrapods in Europe, the loggerhead sea turtle is one of the most protected under international agreements, national legislation, and directives: It is included in Annex A of the Convention on International Trade in Endangered Species of Wild Fauna and Flora (CITES), in Appendix II of the Bonn Convention, in Annex II of the Barcelona Convention, in Annex III of the Berne Convention, and in Annexes II and IV of the Habitats Directive.

Loggerhead sea turtle is globally listed as Vulnerable (VU) in the IUCN Red List of Threatened Species; the global population trend is decreasing due to fragmentation, fluctuations, and a continuous decline of mature individuals (Casale and Tucker 2017; IUCN), while the Italian population is classified as endangered (EN) by the IUCN Italian Red List (Rondinini et al. 2022). Loggerheads are affected by several threats (Tomás et al. 2008; Casale 2010, 2011), including the human‐induced alteration of coastal environments, the disturbance of the nesting sites, fishery, and pollution (Casale and Margaritoulis 2010; Wallace et al. 2011).

The primary objective for the conservation of the species is to identify, protect, and manage the nesting sites; to achieve this goal, Special Areas of Conservation (SACs) have been established in Europe. Nowadays, the species distribution models (SDMs) are widely applied to understand various aspects of ecology, biogeography, and biodiversity conservation in order to investigate the distribution of threatened species (Engler et al. 2004; Bombi et al. 2009; Iannella et al. 2018) like the loggerhead sea turtle.

Therefore, the aims of this study are: (i) to establish and update the list of nesting sites in Sicily and its minor islands; (ii) to investigate the Sicilian distribution and nesting suitability using spatial distribution models (SDMs); (iii) to perform a gap analysis in a GIS environment considering the protected area network in Sicily.

## 2 Materials and Methods

The region of Sicily is located in the centre of the Mediterranean Sea, and it is composed of the largest island of the Mediterranean sea with an area of 25,711 km^2^ and the surrounding 15 minor islands, with an area of 285.4 km^2^. The main island has a remarkable coastline. Of the 1152 km, approximately 550 km (47.8%) consist of beaches and/or dune environments divided into 99 coastal segments (Corine Biotopes code: 16.1 sea beaches and 16.21 Shifting dunes).

Specific data on nesting areas of loggerhead sea turtles were collected through a bibliographic analysis, personal observations, reports from the local Wildlife division or local people witnessing the nesting events, referrals on websites and social networks often related to monitoring activities (WWF's Progetto Tartarughe in Sicily). Duplications (i.e., clutches laid by the same female) cannot be excluded. After georeferencing, all the locations of nests were analyzed by using GIS to realize distribution maps.

Many ecological models predicting the spatial distribution of species have been developed. In order to define the suitability species distribution model (SDM) of *Caretta caretta* in the Sicily main island, therefore excluding minor islands, we used the maximum entropy method implemented through the MaxEnt software (Phillips et al. 2006, 2017; Phillips and Dudík 2008), already used to assess the suitability of foraging areas for *Caretta caretta* (Fujisaki et al. 2020). This method, which has often been acknowledged as one of the best performing modeling algorithms (Elith et al. 2006; Pearson et al. 2007), unlike generalized linear models (GLMs) and generalized additive models (GAMs), which need a real absence data, uses pure machine‐learning techniques for modeling species distributions from presence‐only records (Elith et al. 2006; Phillips et al. 2006; Merow et al. 2013). This perfectly fits our study case, as we are unaware of sites where this species does not nest.

For the construction of the species distribution model pertaining to the major island, we used the nesting records of *Caretta caretta* spanning from 1979 to 2022 (included). The continuous bioclimatic variables from 1979 to 2018, at a 30‐s arc resolution (original resolution ≈1 km^2^ grid cell; average for 1979–2018) were extracted from the Copernicus Climate Change Service's Downscaled Bioclimatic Indicators database for the designated regions (Wouters 2021). The 19 bioclimatic variables selected from the Copernicus database correspond to those reported in the WorldClim database (Table 1), in order to allow a comparison over time.

**TABLE 1:** The dataset of the nineteen bioclimatic variables considered as candidate predictors (from Worldclim.org), with their codes and explication.

BIO1 = Annual Mean Temperature
BIO2 = Mean Diurnal Range (Mean of monthly (max temp—min temp))
BIO3 = Isothermality (BIO2/BIO7) (* 100)
BIO4 = Temperature Seasonality (standard deviation *100)
BIO5 = Max Temperature of Warmest Month
BIO6 = Min Temperature of Coldest Month
BIO7 = Temperature Annual Range (BIO5‐BIO6)
BIO8 = Mean Temperature of Wettest Quarter
BIO9 = Mean Temperature of Driest Quarter
BIO10 = Mean Temperature of Warmest Quarter
BIO11 = Mean Temperature of Coldest Quarter
BIO12 = Annual Precipitation
BIO13 = Precipitation of Wettest Month
BIO14 = Precipitation of Driest Month
BIO15 = Precipitation Seasonality (Coefficient of Variation)
BIO16 = Precipitation of Wettest Quarter
BIO17 = Precipitation of Driest Quarter
BIO18 = Precipitation of Warmest Quarter

*Note:* BIO19 = Precipitation of Coldest Quarter.

The Copernicus dataset contains the average values of the bioclimatic variables between 1979 and 2018. To avoid possible multicollinearity of the 19 environmental variables within the considered years, we performed a correlation test. We assessed the degree of intercorrelation by computing pairwise Pearson's correlation coefficient. Subsequently, we selected the most important factors within pairs of variables with Pearson |*r*| > 0.85 and excluding the ones having less ecological importance to the species based on its autoecology. Strongly correlated variables were excluded to prevent obtaining misshaped results. Therefore, weakly correlated environmental variables were considered to estimate the percentage contribution of each of them to the choice of nesting sites. Five climate variables were selected: Bio05 (maximum daily temperature of the month with the highest monthly mean of daily mean temperature), Bio07 (maximum temperature of the warmest month minus minimum temperature of the coldest month), Bio11 (the mean of monthly mean temperature during the coldest quarter, defined as the quarter with the lowest monthly mean (of the daily mean) temperature using a moving average of 3 consecutive months), Bio12 (annual mean of the daily mean precipitation rate), and Bio13 (maximum of the monthly precipitation rate).

To perform the MaxEnt model, several default parameters were retained unchanged, such as a convergent threshold of 10^−5^, a maximum interaction value of 500, and a maximum of 10,000 randomly selected background points, as recommended by previous studies (Phillips and Dudík 2008; Anderson et al. 2016; Bargain et al. 2017). The predictive model was computed based on 10 replicates for the “Regularization Multiplier” (RM) parameter value. We opted for an RM of 1 to balance data adherence and model complexity and to minimize the risk of overfitting (Phillips et al. 2017; Bargain et al. 2018). For the modeling evaluations, we took into consideration the AUC values, the standard deviation, the TSS mean, and the TH value.

To assess the potential impact of climate change on the presence of nesting suitability sites for *Caretta caretta* , once the environmental suitability model was obtained using the Copernicus dataset, we projected the predictors onto the WorldClim dataset (ver.2.1), containing the average values of the same variables, relating to the period 1979–2000 (Fick and Hijmans 2017). This was done to generate the corresponding environmental suitability map for the species.

The loggerhead sea turtle's nests distribution map and environmental suitability map were overlaid with the Natura 2000 sites map in Sicily, in order to establish how many nests fit into Natura 2000 sites, including Special Protection Areas (SPAs) and Special Conservation Areas (SACs).

## 3 Results

**FIGURE 1:**
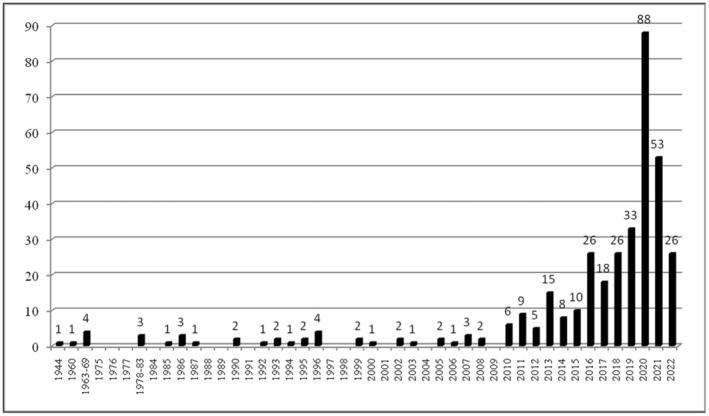
Temporal distribution of loggerhead turtle's nests (*n* = 363) recorded in Sicily (Italy) in the 1944–2022 period.

**FIGURE 2:**
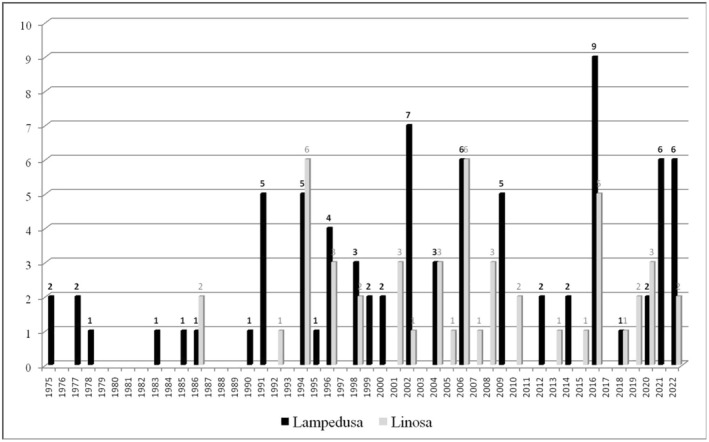
Temporal distribution of loggerhead turtle nests (*n* = 132) recorded in Pelagie Islands (Italy) in the 1975–2022 period.

Between 1944 and 2022, 498 egg depositions occurred in the Sicilian Region (Prato et al. 2022; Table S1): 363 in Sicily (Figure 1), and the remaining 135 in some of its smaller islands. Specifically, 3 egg depositions involved the Eolian Islands (1 in Panarea, 1 in Stromboli, and 1 in Lipari), and 132 the Pelagie Islands (80 in Lampedusa and 52 in Linosa) (Figure 2).

**FIGURE 3:**
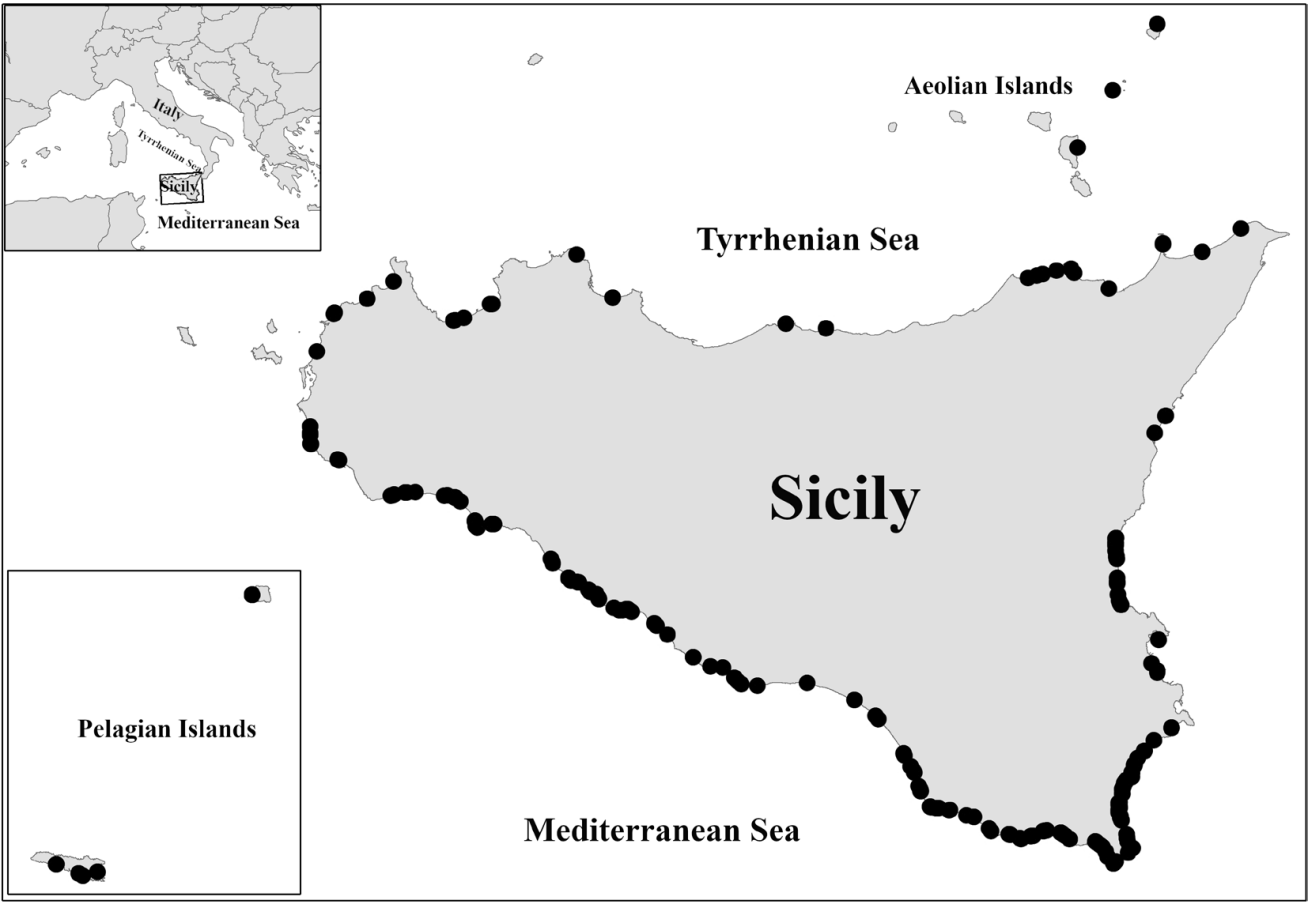
Geographical distribution of egg‐laying sites in Sicily and its smaller islands from 1944 to 2022.

The depositions observed in Sicily have included 99 coastal segments, with varying lengths spanning from 0.4 to 66 km. The majority of the sites are located along the entire southern coast, extending northward along half of the eastern coast of the island (Figure 3).

**FIGURE 4:**
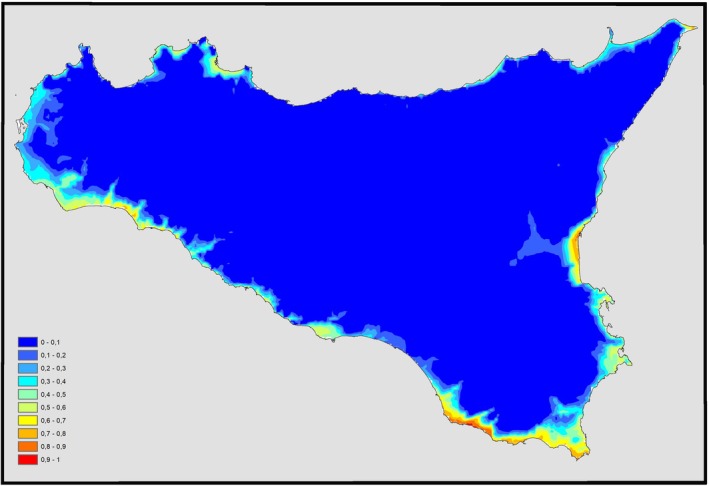
Suitability distribution areas of *Caretta caretta* in Sicily (dataset Copernicus 1979–2018).

About the environmental suitability, according to the classification of Swets (1988), the model of *C. caretta* obtained with the Copernicus dataset (Figure 4) showed very good overall performance (AUC = 0.961; SD = 0.011; threshold = 0.13; TSS = 0.832), thus indicating high predictive power for nest deposition.

The bioclimatic variable with the highest gain when used in isolation is the mean monthly temperature during the coldest quarter (Bio11), with a contribution of 89% (Copernicus). This suggests it provides the most useful information on its own. The contribution of the other considered variables is considerably lower (Table 2).

**TABLE 2:** The selected variables with their percent contribution and permutation importance (dataset Copernicus 1979–2018).

Variable	Percent contribution	Permutation importance
bio11	89	82.8
bio13	7.6	9.6
bio07	5.3	0.8
bio12	3.4	3.9
bio05	2.9	2.9

The resulting map, obtained from the elaboration of nesting data, shows that the maximum value of the suitability estimate (red) by MaxEnt for these areas is equal to 0.97 and how areas of maximum suitability for nesting are mainly along the southern coast of the island, especially the southernmost area, located east of the island. Maximum suitability is also remarkable on the south‐west coast, in the Sicilian Channel (from Mazara del Vallo to Sciacca and Licata), and south of the Ionian slope (from Catania's Gulf to Augusta). Specific areas with a high value of suitability (orange) are displayed along the northern side of the Ionian coast and along the Tyrrhenian coast of the island.

**FIGURE 5:**
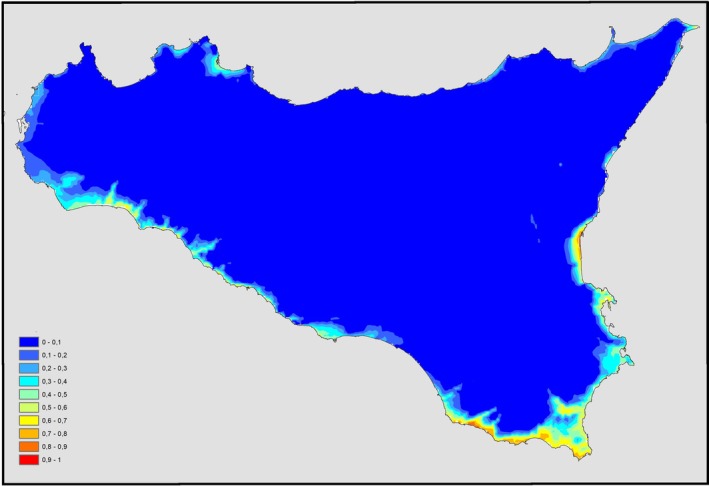
Suitability distribution areas of *Caretta caretta* in Sicily (dataset Worlclim 1979–2000).

Figure 5 shows the model of the environmental suitability realized by projecting the predictive functions obtained with Copernicus onto the WorldClim dataset, which contains the annual averages of the variables for the period 1979–2000. Once again, it showed very good overall performance (AUC = 0.963; SD = 0.011; threshold = 0.164; TSS = 0.904).

Even so, the bioclimatic variable with the highest gain when used in isolation is the mean monthly temperature during the coldest quarter (Bio11), with a contribution of 89.7%, and the contribution of the other considered variables is considerably lower (Table 3). These values are very similar to the ones obtained in the Copernicus dataset (cfr. Table 2).

**TABLE 3:** The selected variables with their percent contribution and permutation importance (dataset Worldsclim 1979–2000).

Variable	Percent contribution	Permutation importance
bio11	89.7	88.2
bio13	3.5	5.2
bio07	2.9	0.2
bio12	2.1	3.2
bio05	2.7	3.2

**FIGURE 6:**
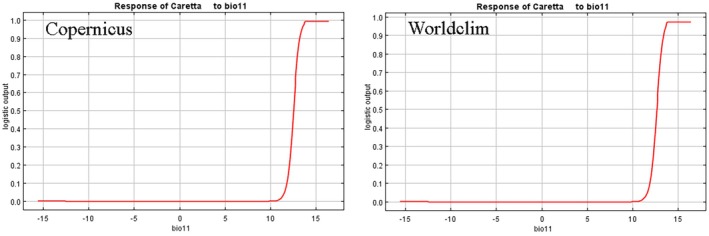
The response curves of the first variable that has the most contribution to the model.

Figure 6 shows how the trend of the environmental variable Bio11 affects the Maxent prediction obtained with the Copernicus and WorldClim datasets.

The maxent suitability map is only based on the occurrence of deposition within the analyzed cells, regardless of the amount of depositions in each of them. Connecting the suitability degree of the cells in which the deposition occurred with the number of depositions known, a significant correlation value emerged (*p* = 0.012).

Comparing the suitability maps (Figures 4 and 5), it is possible to underline how some areas were, at first, less suitable and recently turned into more suitable sites for nesting, due to higher temperatures reached in the latest years, which led to new depositions along the Tyrrhenian coast of Sicily (Figure 3).

**FIGURE 7:**
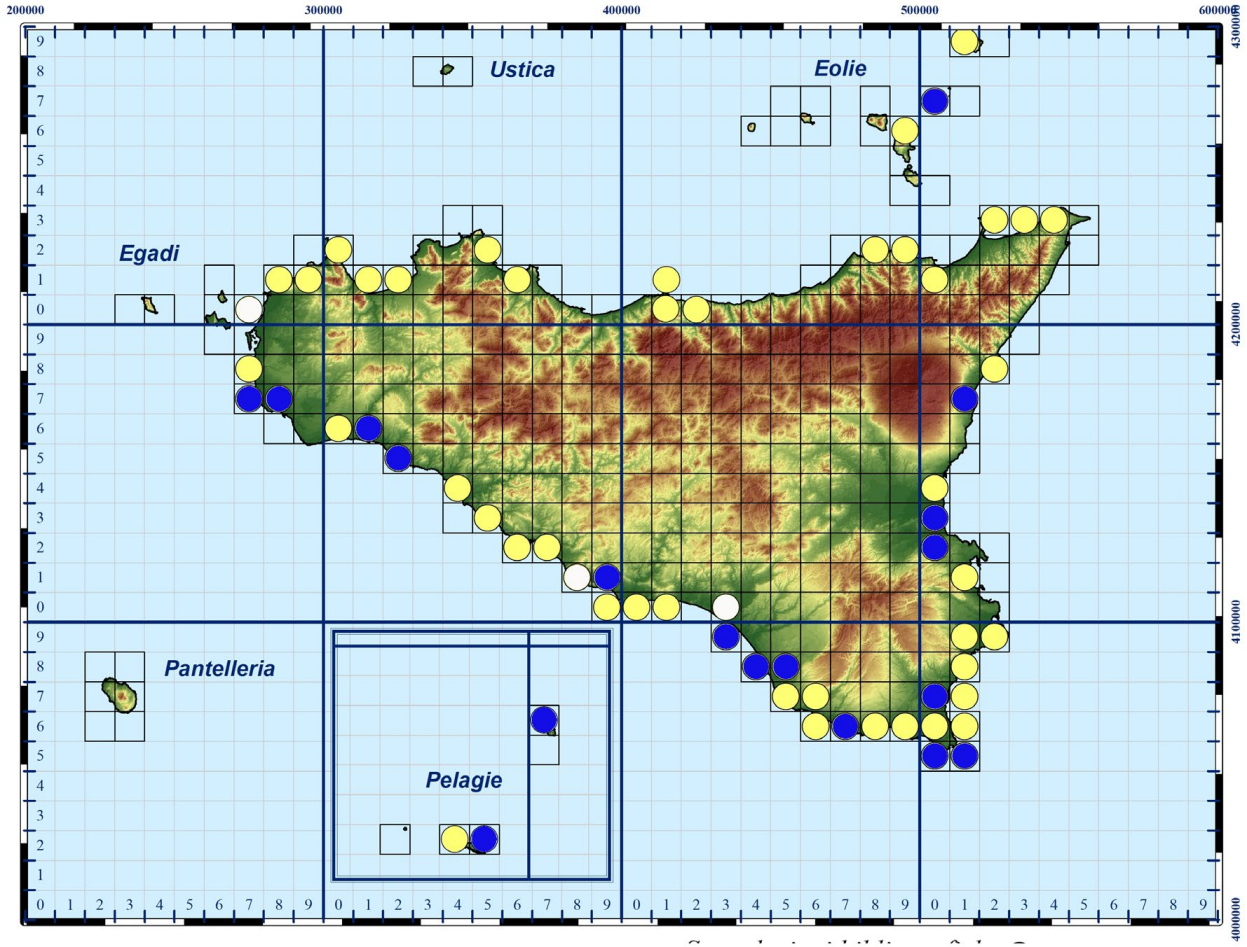
Atlas updated map (UTM WGS84) of *Caretta caretta's* egg‐laying sites in Sicily. Blue circle = confirmed previous atlas data; yellow circle = new data; white circle = non confirmed data.

Out of the 363 depositions that occurred in Sicily from 1944 to 2022, 169 of them affected 22 Natura 2000 sites, specifically 5 Special Protection Areas (SPAs), 2 Special Protection Areas/Special Areas of Conservation (SPA/SAC), and 15 (Special Areas of Conservation (SACs)) (Figure 7). Among these, only 8 sites (36.4%) reported *Caretta caretta* within the standard data form, which nevertheless refers to the protection of the species in relation to emerged areas. The remaining 194 (53%) depositions occurred outside of these sites: in some cases, the nests are very close to the edges of the Natura 2000 sites, although a considerable number (*n* = 77) of depositions occurred far from any of these sites.

Figure 7 shows UTM Map (gridcell 10x10km) update of *C. caretta* in Sicily, based on previous distribution atlases (Turrisi and Vaccaro 1998; Lo Valvo and Longo 2001; Bella and Turrisi 2005; AA.VV. 2008; Giacoma et al. in Corti et al. 2010), new literatures and online sources, including depositions in 2022.

## 4 Discussion

Nesting events are known for Sicily since the XIX century (Doderlein 1871). The first distribution map of *Caretta caretta*, concerning the period between 1975 and 1997 and based on nesting events, defines Sicily as an irregular nesting site (Turrisi and Vaccaro 1998), while the nesting events in Lampedusa and Linosa islands are classified as regular nesting sites (Turrisi and Vaccaro 1998; Lo Valvo and Longo 2001; Bella and Turrisi 2005; AA.VV 2008; Giacoma et al., in Corti et al. 2010). The produced map of Sicily (UTM WGS84; gridcell 10×10 km^2^) shows the presence of reproductive sites within 7 gridcells. A second map, corresponding to the period 1975–1999, reports instead the presence of reproductive sites falling in 6 gridcells (Giacoma et al. in Corti et al. 2010). The Italian herpetological atlas (Sindaco et al. 2006), updated to 2005, strangely did not consider the species as a nesting population on the island. A further distribution map, taking into account the nests observed between 1980 and 1999, reports 13 gridcells occupied, which are reduced to 9 by 2006 (AA.VV 2008).

From 2011, the number of nesting events has shown a significant increase compared to the past, and it appears to exhibit regular patterns over time, especially along the southern coast of the island (Casale et al. 2012). A very lacking map concerning 25 depositions in Sicily for the years 2017 and 2018 has been published by Surdo and Massa (2020), although the nesting events known for this same period are 37 (cf. Prato et al. 2022). A more comprehensive map is provided by Prato et al. (2022), which covers depositions from 1944 to 2021.

This abrupt increase of nests and colonizing individuals (cfr. Luna‐Ortiz et al. 2024) in the past decade has been interpreted as the result of heightened beach monitoring efforts, associated with the activation and implementation of LIFE projects, as well as increased public awareness. Citizens have learned to recognize the tracks left on the sand by female turtles during their excursions for nesting and they report their presence to authorities (Pietroluongo et al. 2021). Furthermore, it is likely due to an actual increase in the number of nestings in Sicily, contrary to the statement made by Surdo and Massa (2020). This increase, which seems to be confirmed by nesting events on beaches characterized by high and constant human activities, where the presence of the species had never been reported before, is also highlighted by the increasing number of sea turtle rescues at marine turtle rehabilitation centers in recent years (cf. Caracappa et al. 2018), but also by the increasing number of sightings at sea and along the island's coasts.

The hypothesis of an increase in the breeding sites in Sicily is also supported by the comparison of the two suitability maps obtained for different periods.

It is reasonable to assume that the demographic increase of nesting events on the island is induced by the northward shift of the species, due to the increase in temperatures and the presence of beaches that have become suitable for nesting. Just at the beginning of the nesting season, the Tyrrhenian coast of Sicily already counts twelve nests only in the province of Palermo (Mondello Beach, Trappeto, Cefalù; Pozzillo, Balestrate, Isola delle Femmine, Lascari; source: firsthand data from WWF Italia). Like many thermophilic species (cfr. Ventura et al. 2019; Esposito et al. 2021; Iveša et al. 2021), loggerhead turtles could use warmer waters and enlarge their habitat to avoid competition for food and breeding. Northward displacement is likely to be facilitated by climate change and rising sea surface temperatures (SST) in recent years (Pastor et al. 2018; Reddin et al. 2022; Hamdeno and Alvera‐Azcarate 2023). In support of this theory, the unusual but increasingly common nests is observed in the northern Tyrrhenian and northern Adriatic (Hochscheid et al. 2022).

Therefore, Sicily has now become not only a regular site for loggerhead sea turtle oviposition but also an important area for the conservation of this turtle species in the central Mediterranean region. Consequently, it is essential to protect these new nesting sites through the implementation of already existing conservation measures and the institution of new conservation measures. Despite this increase, an update has not yet been undertaken.

In consideration of the importance of Sicily today as an egg‐laying area for *Caretta caretta* , it would be essential to update the ecological network for the protection of this species, including, if not present, the taxon in the standard form of sites already established, modifying the boundaries of some sites already established by expanding their surfaces or establishing new ones by including areas suitable for deposition and inserting the species in the standard forms (Table 4).

**TABLE 4:** Suggested updates to sites of community importance for a better ecological network for the protection of *Caretta caretta* in Sicily.

	Present	Newly established	Extension	Form	
ITA010011	SAC		X		Including the beach to the east up to the mouth of the Cavarretto torrent (UTM WGS84 33S 320202, 4159140)
ITA040003	SAC		X		Including the beach to the east up to Punta secca (UTM WGS84 33S 359700, 4130940)
ITA040015	SAC		X		Including the beach to the east up to Porto Empedocle harbor (UTM WGS84 33S 369130, 4127660)
ITA030032	SAC		X	X	Including the beach to the south (Angonia's Beach)
ITA070001	SPA	SAC	X	X	
ITA070029	SPA	SAC		X	
ITA070030	SAC			X	
ITA090002	SAC			X	
ITA090003	SAC			X	
ITA090004	SAC			X	
ITA090010	SAC			X	

Eventually, it would be necessary to establish a new SAC coinciding with the Calabernardo Beach (Lido di Noto, Siracuse).

## Author Contributions

**Chiara Siddiolo:** conceptualization (equal), data curation (equal), methodology (equal), supervision (equal), validation (equal), writing – original draft (equal), writing – review and editing (equal). **Antonietta Rosso:** data curation (equal), supervision (equal), validation (equal), writing – original draft (equal). **Grazia Orecchio:** data curation (equal), formal analysis (equal), visualization (equal). **Mario Lo Valvo:** conceptualization (equal), data curation (equal), formal analysis (equal), methodology (equal), software (equal), supervision (equal), validation (equal), visualization (equal), writing – original draft (equal), writing – review and editing (equal).

## Conflicts of Interest

The authors declare no conflicts of interest.

## Supporting information


Table S1.

## Data Availability

Data are available from the Dryad Digital Repository: http://datadryad.org/stash/share/uTRCjnkxKSWbVzZYJMHS8zvr8US2uEIL2ljZJzQD0zQ.
