# Supplementary material for: Heat Nests: The Impact of Climate Change on Loggerhead Turtle (Caretta caretta) Nesting Distribution in Sicily (Italy)
Source: Ecol Evol. 2025 Apr 21;15(4):e71177. doi: 10.1002/ece3.71177 (PMC12011450; doi:10.1002/ece3.71177)
Supplement: Supplementary file 1 — Table S1. [file ECE3-15-e71177-s001.docx]

| **Year** | **Province** | **Locality** | **N** | **Reference** |
| --- | --- | --- | --- | --- |
| 2013 | Trapani | Triscina - Spiaggia Gaggera | 1 | Faraone Francesco Paolo (pers.comm.) |
| 2014 | Agrigento | Torre Salsa | 2 | https://www.strettoweb.com/2014/09/agrigento-schiuse-22-uova-tartaruga-caretta-caretta/183323/ |
| 2018 | Caltanissetta | Gela | 1 | https://www.nuovosud.it/79673-cronaca-caltanissetta/schiuse-uova-di-tartaruga-caretta-caretta-spiaggia-gela |
| 2022 | Agrigento | Lampedusa | 6 | https://tartapedia.it/ |
| 2022 | Agrigento | Licata, Mandy Beach | 1 | https://tartapedia.it/ |
| 2022 | Agrigento | Linosa | 2 | https://tartapedia.it/ |
| 2022 | Agrigento | Menfi, Contrada Cipollazzo | 1 | https://tartapedia.it/ |
| 2022 | Agrigento | Siculiana Marina | 1 | https://tartapedia.it/ |
| 2022 | Caltanissetta | Gela | 1 | https://tartapedia.it/ |
| 2022 | Catania | Oasi del Simeto | 3 | https://tartapedia.it/ |
| 2022 | Messina | Gioiosa Marea | 3 | https://tartapedia.it/ |
| 2022 | Messina | Laghetti di Marinello, Patti | 1 | https://tartapedia.it/ |
| 2022 | Ragusa | Punta Secca, S.C. Camerina | 1 | https://tartapedia.it/ |
| 2022 | Ragusa | Scoglitti, Vittoria | 6 | https://tartapedia.it/ |
| 2022 | Siracusa | Agnone Bagni, Augusta | 1 | https://tartapedia.it/ |
| 2022 | Siracusa | Isola delle Correnti | 5 | https://tartapedia.it/ |
| 2022 | Siracusa | Noto, Vendicari | 1 | https://tartapedia.it/ |
| 2022 | Siracusa | Priolo Gargallo, Saline | 1 | https://tartapedia.it/ |
| 2022 | Trapani | Alcamo Marina | 1 | https://tartapedia.it/ |

| FP Faraone comm pers. |
| --- |
| https://www.strettoweb.com/2014/09/agrigento-schiuse-22-uova-tartaruga-caretta-caretta/183323/ |
| https://www.strettoweb.com/2014/09/agrigento-schiuse-22-uova-tartaruga-caretta-caretta/183323/ |
| <https://www.nuovosud.it/79673-cronaca-caltanissetta/schiuse-uova-di-tartaruga-caretta-caretta-spiaggia-gela> |
